# Biosynthesis of Platinum Nanoparticles with Cordyceps Flower Extract: Characterization, Antioxidant Activity and Antibacterial Activity

**DOI:** 10.3390/nano12111904

**Published:** 2022-06-02

**Authors:** Ling Liu, Yun Jing, Ailing Guo, Xiaojing Li, Qun Li, Wukang Liu, Xinshuai Zhang

**Affiliations:** 1 College of Food Science and Technology, Huazhong Agriculture University, Wuhan 430070, China; 18438605237@163.com (L.L.); liqun0617@126.com (Q.L.); liu_wukang@126.com (W.L.); hnzhangxinshuai@163.com (X.Z.); 2 Kaibei Technology (Suzhou) Co., Ltd., Suzhou 215000, China; yj@capernano.com (Y.J.); xiaojing.li@capernano.com (X.L.)

**Keywords:** cordyceps flower, platinum nanoparticles, biosynthesis, characterization, antioxidant activity, antibacterial activity

## Abstract

The aim of this work is to develop a green route for platinum nanoparticles (PtNPs) biosynthesized using Cordyceps flower extract and to evaluate their antioxidant activity and antibacterial activity. Different characterization techniques were utilized to characterize the biosynthetic PtNPs. The results showed that PtNPs were spherical particles covered with Cordyceps flower extract. The average particle size of PtNPs in Dynamic Light Scattering was 84.67 ± 5.28 nm, while that of PtNPs in Transmission Electron Microscope was 13.34 ± 4.06 nm. Antioxidant activity of PtNPs was evaluated by DPPH free radical scavenging ability test. The results showed that the antioxidant activity was positively correlated with the concentration of PtNPs, the DPPH scavenging efficiency of PtNPs (0.50–125.00 μg/mL) was 27.77–44.00%. In addition, the morphological changes of four kinds of bacteria (*Escherichia coli*, *Salmonella typhimurium*, *Bacillus subtilis*, *Staphylococcus aureus*) exposed to PtNPs were observed by scanning electron microscope. The results showed that the antibacterial activity of PtNPs against Gram-negative bacteria was stronger than that of Gram-positive bacteria.

## 1. Introduction

In recent years, platinum nanoparticles (PtNPs) have become a hot research topic due to their unique antibacterial [1,2], anticancer [3,4], catalytic [5] and antioxidant activities [6]. The conventional preparation methods of PtNPs mainly include physical and chemical methods. In physical methods, mechanical pressure, high-energy radiation, thermal energy or electrical energy are used to gradually reduce the size of the material to nanometer scale [7], which requires higher energy, temperature or pressure. In chemical methods, electrochemical, chemical reduction, or photochemical reduction techniques are used to reduce metal cations to nano-scale metal monomers. Harsh synthetic conditions, toxic chemicals and organic solvents [8,9] are often involved in this process, which can be harmful to human beings and the environment. Therefore, the development of biosynthesis methods of PtNPs has become an important aspect of nanomaterials research.

The biosynthesis process mainly involves plants, bacteria and fungi. It has been reported that the synthesis of metal nanoparticles from plant extracts is faster than that from fungi and bacteria [10]. Plant extract-mediated biosynthesis of nanoparticles has become an important research field of modern nanotechnology because of its simplicity and eco-friendliness.

Cordyceps flower is the fruiting body of artificial *Cordyceps militaris* belonging to *Cordyceps*, Clavicipitaceae, Ascomycotina, which is formed by fungus inoculation on nutrient sufficient medium. The current research reports on the application of Cordyceps flower are mainly focused on the optimization of the extraction process of chemical components, including polysaccharides, ergosterol and total flavonoids [11,12,13] and biomedical applications, including prevention of hyperlipidemia, anticancer aspects [14,15,16]. It is worth noting that some articles have reported the use of *Cordyceps militaris mycelium* to biosynthesize metal nanoparticles, such as silver nanoparticles, gold nanoparticles, zinc oxide nanoparticles [17,18,19]. Similar chemical compositions can be found in Cordyceps flower and *Cordyceps militaris mycelia* [20,21], but there is no report on the biosynthesis of PtNPs using Cordyceps flower extract.

Therefore, the objective of this study is the biosynthesis of PtNPs using Cordyceps flower extract under heating and stirring conditions. The synthesized PtNPs were characterized by a variety of characterization techniques to explore their morphological, physical and chemical properties. Then, the antioxidant activity of PtNPs was evaluated by DPPH free radical scavenging ability test. In addition, the morphological changes of four kinds of bacteria before and after exposure to PtNPs were observed by scanning electron microscope to evaluate the antibacterial activity of biosynthetic PtNPs.

## 2. Materials and Methods

### 2.1. Materials

Hexachloroplatinic acid (H_2_PtCl_6_·6H_2_O) was purchased from Shanghai Macklin Biochemical Co., Ltd. (Shanghai, China). Cordyceps flower was purchased from Huangniwa Town, (Liaoyang, China). DPPH free radical scavenging kit was purchased from Nanjing Jiancheng Institute of Biological Engineering (Nanjing, China). All the bacterial strains were provided by Wuhan Center for Disease Control and Prevention (Wuhan, China).

### 2.2. Methods

#### 2.2.1. Preparation of Cordyceps Flower Extract

The Cordyceps flower was dried at 5 °C and ground into fine powder. For the aqueous extraction, 10.0 g of fine powder was extracted with 100 mL distilled water at room temperature for 30 min in water bath. The supernatant retained after centrifugation (11,000 r/min, 30 min) was filtered by the cellulose acetate membrane with pore diameter of 0.22 μm to obtain sterile Cordyceps flower extract (CE). The CE was stored at 4 °C and should be used as soon as possible.

#### 2.2.2. Synthesis of PtNPs

According to previous experimental exploration and literature reports, an alkaline environment is more conducive to the preparation of stable and uniform PtNPs solution [22,23]. The CE was adjusted from the original pH 6.0 to the pH 10.0, and then 50 mL CE was added to 50 mL H_2_PtCl_6_ (1 mmol/L) aqueous solution. The mixture was heated at 50 °C and stirred at high speed for 1 h. The color change from pale yellow to dark brown of the mixture indicated the formation of PtNPs. The product was collected by centrifugation at 4000× *g* r/min for 10 min and washed three times with distilled water to remove the unreacted substances. The obtained nanoparticles were freeze-dried in vacuum and then ground into powder for further analysis.

#### 2.2.3. Characterization of PtNPs

Ultraviolet-visible spectroscopy (UV-vis) (Shimadzu Suzhou Instruments Mfg. Co., Ltd., Suzhou, China) was used to identify whether PtNPs were successfully synthesized. Using distilled water as blank control, PtNPs and CE were scanned in the wavelength range of 200–800 nm. The particle size, polydispersity index (PDI) and zeta potential of PtNPs in liquids were measured by dynamic light scattering (DLS) (Malvin Instrument Co., Ltd., Shanghai, China). The morphology and elemental composition of PtNPs were characterized by Field emission scanning electron microscope (FE-SEM) (Tianmei China Science Instrument Co., Ltd., Beijing, China) and energy dispersive X-ray spectroscopy (EDS) (Tianmei China Science Instrument Co., Ltd., Beijing, China). The morphology of PtNPs was further observed by transmission electron microscope (TEM) (Hitachi Science Instrument (Beijing) Co., Ltd., Beijing, China). The diffraction pattern of the samples was determined by X-ray diffractometer (XRD) (Bruker (Beijing) Technology Co., Ltd., Beijing, China). The scanning range of 2θ was from 10° to 90° with a scan rate of 8°/min. Fourier-transform infrared spectroscopy (FT-IR) (Thermo Nicolet Corporation, Beijing, China) was used in order to determine the functional groups present in the CE and synthesized PtNPs. The dried PtNPs were mixed with KBr at a mass ratio of 1:100 and then thoroughly ground and pressed into thin slices. Infrared absorption spectra within the range 4000–400 cm^−1^ at a 4 cm^−1^ resolution using 32 scans were recorded at room temperature.

#### 2.2.4. Evaluation of Antioxidant Activity

DPPH free radical scavenging ability kit was used to evaluate the antioxidant activity of PtNPs. According to the volume ratio of 2:3, the samples mixed with DPPH working solution were labeled as test group, the samples mixed with 80% methanol were labeled as control group, and 80% methanol mixed with DPPH working solution was labeled as blank group. Each group was placed at room temperature in dark for 30 min, and then the absorbance at 517 nm was measured and recorded. Similarly, different concentrations of Trolox solutions were mixed with DPPH working solution to prepare standard curves. The lower the absorbance of the reaction mixture, the higher the free radical scavenging activity. The calculation formula of DPPH free radical scavenging rate is as follows:

(1) $$DPPH\;scavenging\;effect\;\left(\%\;inhibition\right)=\left(1-\left(A_{1}-A_{2}\right)÷A_{0}\right)\times 100\%$$

where *A*_1_ represents the absorbance of test group, *A*_2_ represents the absorbance of control group and *A*_0_ represents the absorbance of blank group. The experiment was repeated three times and the average value was calculated.

#### 2.2.5. Evaluation of Antibacterial Activity

Four kinds of bacterial suspension (10^7^ CFU/mL) were centrifugated at 4000 r/min for 10 min at 4 °C. Following centrifugation, the supernatant was removed, and the bacterial pellet was washed with PBS (0.02 mol/L, pH 7.4) three times. After the precipitate was resuspended in 500 μL PBS, equal volume of PtNPs (100 μg/mL) was added in the test group, and equal volume of PBS was added in the control group. After being resuspended at room temperature for 15 min, these groups were centrifugated at 4000 r/min for 10 min at 4 °C. After being washed twice with PBS, the precipitate was resuspended in 2.5% glutaraldehyde (25% glutaraldehyde:distilled water:0.2 mol/L PBS = 1:4:5) and then fixed at 4 °C for 12 h. The supernatant was removed, and the precipitate was washed with PBS for three times. Then, gradient elution was carried out with ethanol with volume fractions of 30%, 50%, 70%, 90% and 100%. Each step involved resuspending for 10 min and then centrifuging at 4000× *g* r/min for 10 min at 4 °C. The precipitate was resuspended in 100% tert-butanol at 4 °C for 30 min and then freeze-dried in vacuum.

A little freeze-dried bacterial powder was evenly attached to the conductive tape. After spraying gold in a vacuum coating machine, the bacterial morphology of the test group and the control group were observed by FE-SEM. Each sample was photographed and several representative pictures were saved.

## 3. Results and Discussion

### 3.1. Characterization of PtNPs

#### 3.1.1. UV-vis and FT-IR Spectral Analysis

**Figure 1:**
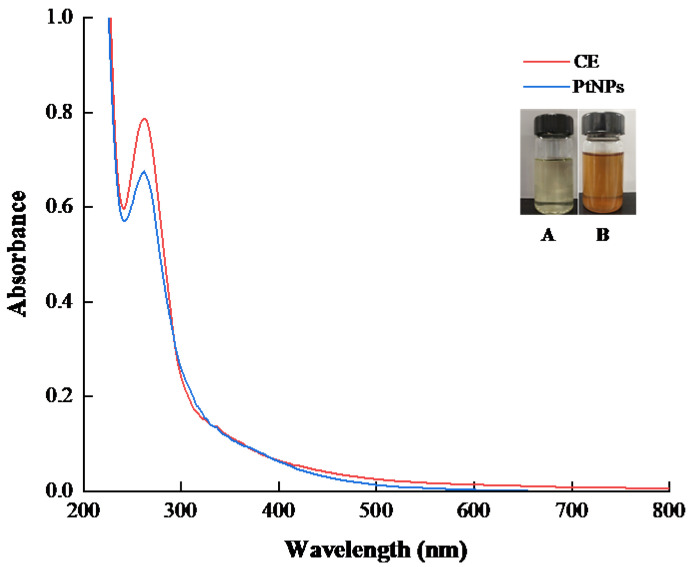
UV-vis spectra of CE and PtNPs. The inset figure shows the color changes of PtNPs formation: (**A**) a mixture of Hexachloroplatinic acid and CE; (**B**) a mixture of the remaining CE and the synthetic PtNPs.

The UV-vis spectra of CE and PtNPs are shown in Figure 1. The maximum wavelength of CE and PtNPs in the absorption spectra was 260 nm, which was consistent with the experimental results of Jeyapaul U et al. [24], confirming the formation of PtNPs. The reason for the similar absorption peaks may be that CE was coated on the surface of PtNPs as a stabilizer during the reduction of platinum ions to platinum atoms.

**Figure 2:**
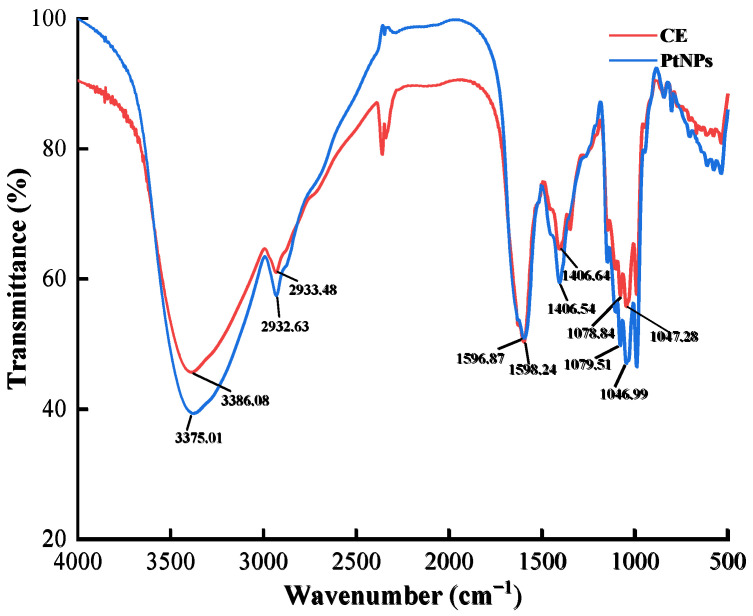
FT-IR spectra of CE and PtNPs.

The FT-IR spectra of CE and PtNPs are shown in Figure 2. The former peaked at 3386.08, 2933.48, 1598.24, 1406.64, 1078.84, 1046.99 cm^−1^, and the latter at 3375.01, 2932.63, 1596.87, 1406.54, 1079.51, 1047.28 cm^−1^, respectively. The dominant peaks in the spectrum of CE also appeared in PtNPs, and the shifts were small. These peaks were derived from the O-H stretching vibration, C-H stretching vibration, -COO- antisymmetric and symmetrical stretching vibration, C-C skeleton vibration, and C-OH stretching vibration, respectively. According to these results, it can be speculated that the functional groups in the PtNPs came from some active substances in CE, such as proteins and polysaccharides, which played a role in reduction and stabilization [25].

#### 3.1.2. DLS Analysis

**Figure 3:**
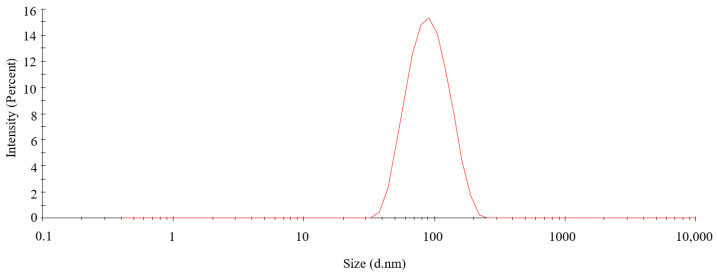
Particle size distribution curve of PtNPs.

**Figure 4:**
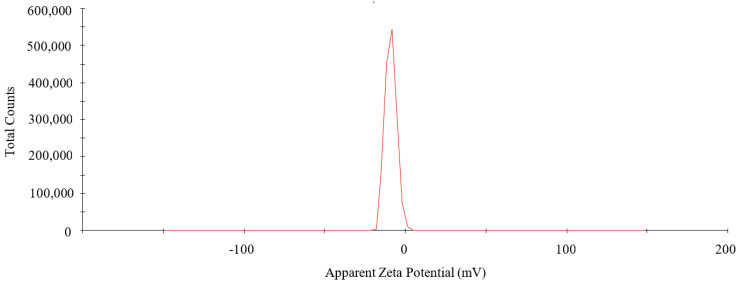
Zeta potential of PtNPs.

The particle size distribution curve and zeta potential of biosynthetic PtNPs are shown in Figure 3 and Figure 4, respectively. The particle size distribution of PtNPs was moderate, and the particles with hydrodynamic diameter of about 100 nm were in the majority. The specific analysis result of Marvin Nano ZS software was that the average particle size of PtNPs was 84.67 ± 5.28 nm, and the PDI value was 0.160, which indicated that synthesized nanoparticles were well monodispersed.

As shown in Figure 4, the zeta potential of PtNPs was −8.64 mV and the peak area intensity was 100%. A negative zeta potential suggested that PtNPs were covered by negatively charged biomolecules, which generated electrostatic repulsion between nanoparticles and reduced the aggregation of nanoparticles, thus increasing the stability of PtNPs [26].

#### 3.1.3. FE-SEM and TEM Analysis

**Figure 5:**
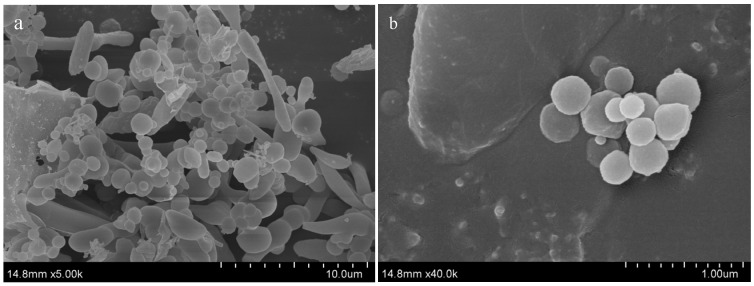
FE-SEM images of PtNPs: (**a**) the field of view at 10 μm; (**b**) the field of view at 1 μm.

The FE-SEM images are shown in Figure 5. Particles with different shapes including larger plant extract particles and spherical PtNPs can be observed in the field of vision. The particle size of PtNPs varied widely, ranging from a small of tens of nanometers to a large of 1 μm. The particle size distributions obtained from FE-SEM and DLS were different, which can be explained by the different sample preparation methods, and the particle agglomeration during the freeze-drying process [27].

**Figure 6:**
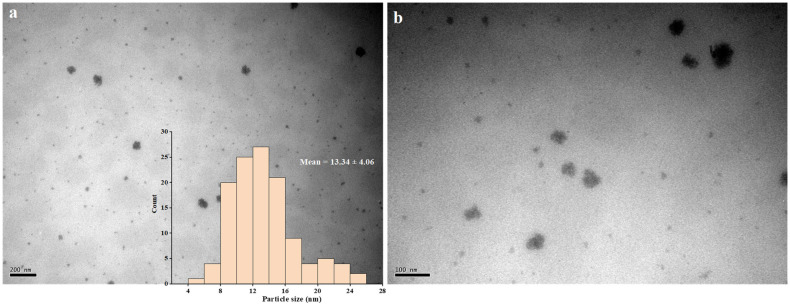
TEM images of PtNPs: (**a**) the field of view at 200 nm; (**b**) the field of view at 10 nm.

The TEM images of PtNPs are shown in Figure 6. It was confirmed once again that the synthesized PtNPs were monodispersed spherical particles. Only a small number of aggregated nanoparticles were observed in the field of vision, while the average particle size was 13.34 ± 4.06 nm. According to the determination principle, these data were closer to the real particle size of PtNPs in the reaction solution [28].

#### 3.1.4. EDS Analysis

**Figure 7:**
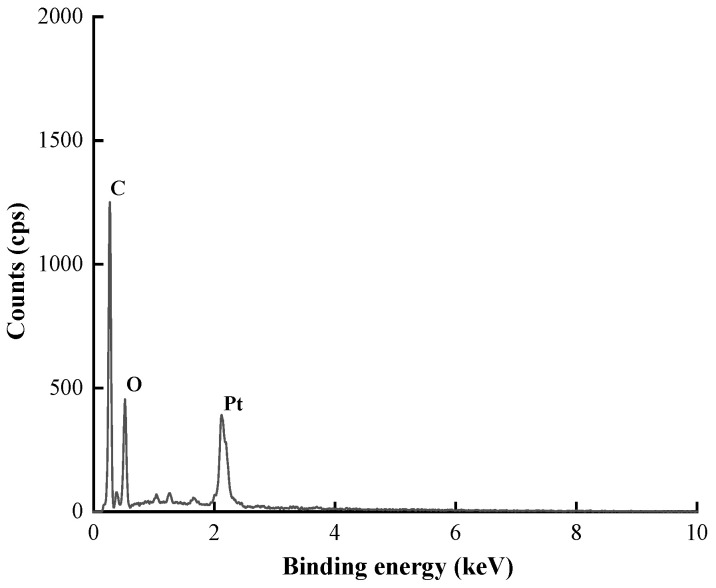
EDS spectrum of PtNPs.

Figure 7 shows the EDS energy spectrum of biosynthetic PtNPs, which mainly involved the peaks of three elements, namely, C, O and Pt. The elemental compositions and proportions on the surface of nanoparticles are listed in Table 1, including Pt (2.41 wt.%, 0.21 at.%), C (47.32 wt.%, 65.96 at.%) and O (20.00 wt.%, 20.93 at.%). The element Au came from the gold plating pretreatment of the sample before observation. Based on the above results, it is speculated that the samples were nanoparticles obtained by the combination of plant extract and metal [29].

#### 3.1.5. XRD Analysis

**Figure 8:**
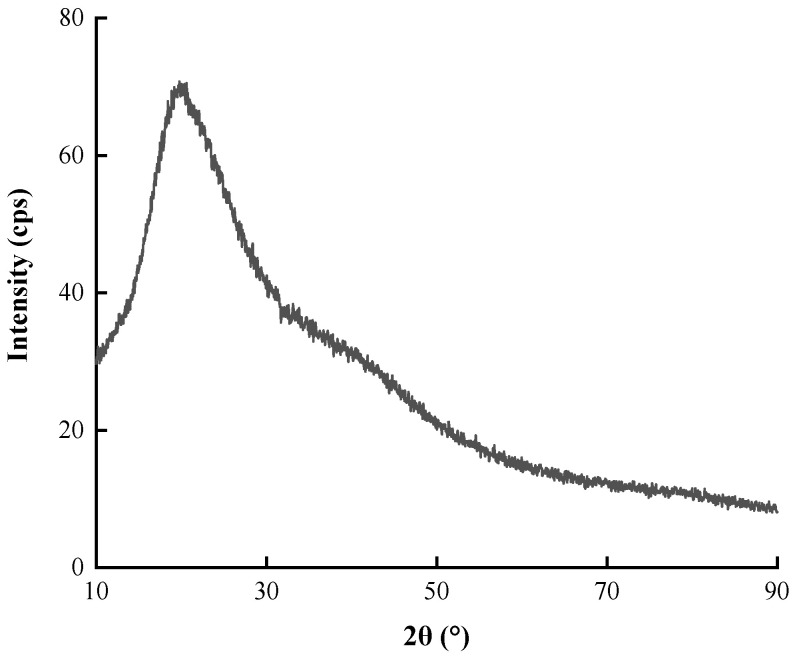
XRD spectrum of PtNPs.

Figure 8 shows the XRD spectrum of PtNPs biosynthesized using CE. A relatively high intensity diffuse packet was observed near 2θ = 25°, which corresponded to the characteristic diffraction peak of organic matter. The lack of characteristic diffraction peak of PtNPs crystal indicated that the synthesized PtNPs were mainly amorphous materials [30]. The reason is probably that the surface of PtNPs was covered by organic compounds in plant extracts, resulting in the masking of the diffraction peak of PtNPs, which can be demonstrated by Deng Zhenning’s research results of the synthesis of iro nanoparticles using the reducing component of sunflower [31].

### 3.2. Antioxidant Activity of PtNPs

**Figure 9:**
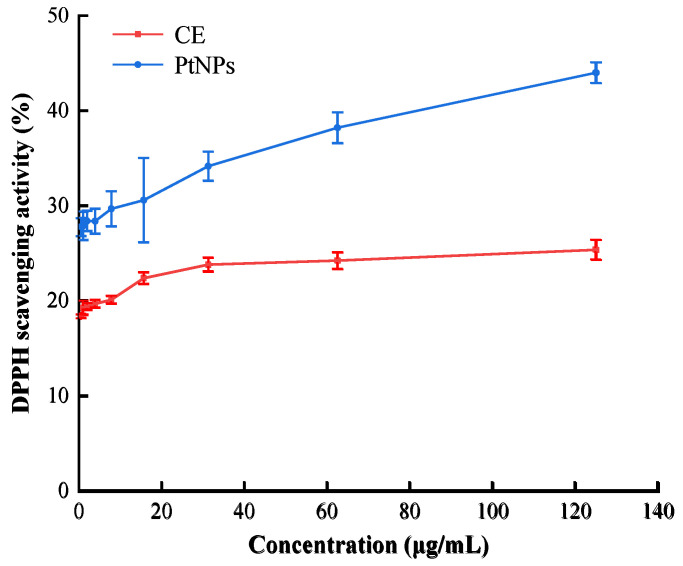
DPPH scavenging activity of PtNPs and CE.

**Figure 10:**
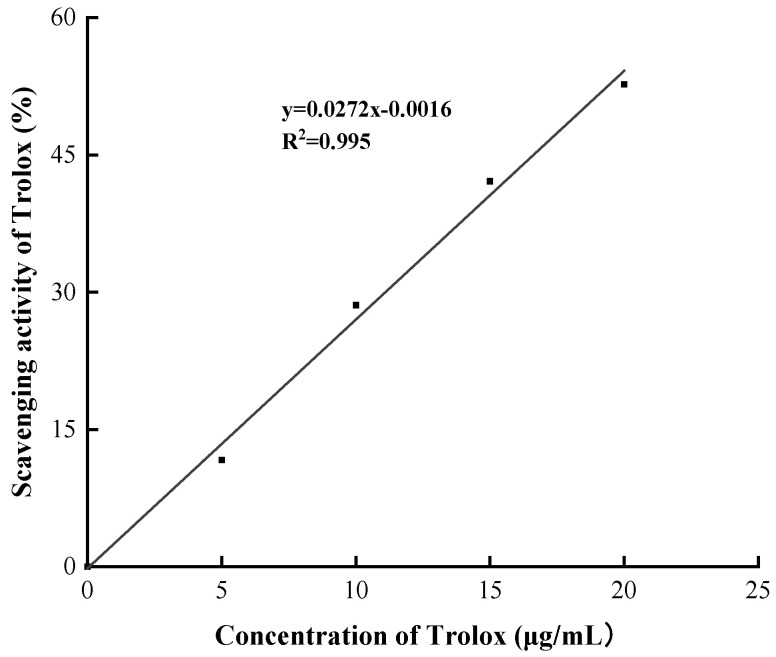
DPPH scavenging activity of standard Trolox.

The scavenging rates of DPPH free radical with different concentrations of pure CE and PtNPs were measured to evaluate the antioxidant activity of the synthesized PtNPs. As shown in Figure 9, with the increase of concentration of samples from 0.50 μg/mL to 125.00 μg/mL, the color change (from purple to light yellow) of DPPH working solution became more obvious, and the DPPH free radical scavenging rate of PtNPs increased from 27.77% to 44.00%, while the scavenging rate of CE increased from 18.41% to 25.37%. The scavenging rate of PtNPs was much higher than that of CE at the same concentration. Additionally, the antioxidant activity of PtNPs was found to be positively correlated with the concentration of PtNPs. Compared with the standard curve in Figure 10, 0.50 μg/mL PtNPs was equivalent to 10.27 μg/mL standard Trolox, while CE of the same concentration was only equivalent to 6.83 μg/mL standard Trolox, indicating that the synthesized PtNPs had significant antioxidant activity. These results were consistent with previous studies on antioxidant activity of PtNPs synthesized using plants [32].

### 3.3. Antibacterial Activity of PtNPs

**Figure 11:**
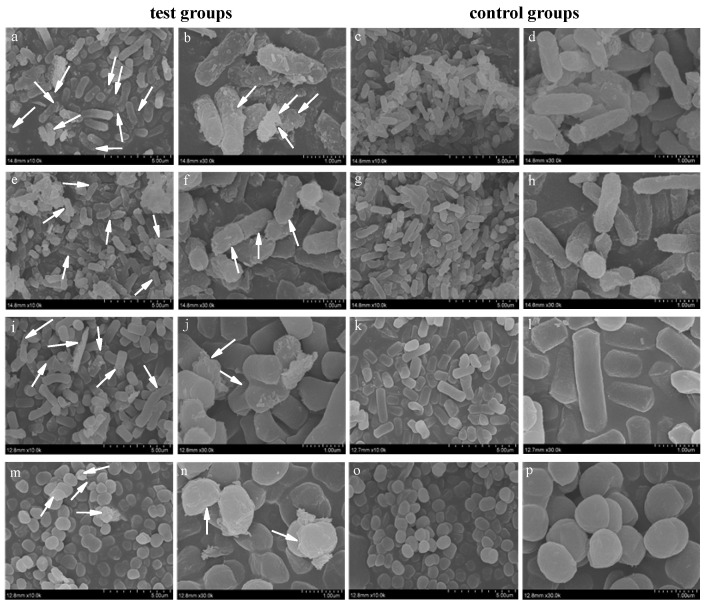
Morphological changes of different bacteria before and after PtNPs treatment: (**a**–**d**) *E. coli*; (**e**–**h**) *S. typhimurium*; (**i**–**l**) *B. subtilis*; (**m**–**p**) *S. aureus*.

Figure 11 shows the morphological changes of Gram-negative bacteria (*E. coli* and *S. typhimurium*) and Gram-positive bacteria (*S. aureus* and *B. subtilis*) observed under FE-SEM after exposure to 100 μg/mL PtNPs solution for 15 min. The bacteria in the control group treated with PBS remained normal rod or spherical, and the outer membranes of bacteria were intact. In contrast, the surfaces of bacteria in the test groups treated with PtNPs showed different degrees of depression, perforation and rupture (as shown by arrow). Cell debris and leaked intracellular matter were observed around the damaged bacteria. By comparing the morphological changes of four kinds of bacteria, it was found that the destructive effect of PtNPs on Gram-positive bacteria was weaker than that of Gram-negative bacteria, which was consistent with the research results of Selvi AM et al. [32].

## 4. Conclusions

In this study, the synthesis of PtNPs through the green chemistry approach has several advantages over conventional chemical methods, as it is simple, fast, inexpensive, with low environmental impact. Specifically, biological resources such as Cordyceps flower extract were employed as both reducers and stabilizers, and universally acceptable solvents such as water were used in this biosynthesis method, which avoided the use of many toxic chemicals. The characterization results showed that PtNPs were spherical particles covered with plant extract. The average particle size of PtNPs in DLS was 84.67 ± 5.28 nm, while that of PtNPs in TEM was 13.34 ± 4.06 nm. In addition, bioactivity assay showed that the antioxidant activity of PtNPs was positively correlated with the concentration of PtNPs, and the destructive effect of PtNPs on Gram-positive bacteria was weaker than that of Gram-negative bacteria. The overall results suggested interesting developments of biosynthesized PtNPs in the fields of antioxidant and antibacterial. Therefore, in the future, further studies will be required for their characterization and possible biomedical applications, including diagnostic, anticancer and antibacterial applications.

## Figures and Tables

**Table 1:** EDS data of PtNPs.

No.	Element	Weight %	Atomic %
1	C K	47.32	65.96
2	N K	9.31	11.13
3	O K	20.00	20.93
4	Pt M	2.41	0.21
5	Au M	20.96	1.78

## Data Availability

Not applicable.
